# Allergic reactions to milk appear sooner than reactions to hen’s eggs: a retrospective study

**DOI:** 10.1186/s40413-016-0104-5

**Published:** 2016-04-11

**Authors:** Noriyuki Yanagida, Takanori Minoura, Setsuko Kitaoka

**Affiliations:** Department of Pediatrics, National Hospital Organization, Sendai Medical Center, Miyagi, Japan; Department of Pediatrics, Sagamihara National Hospital, 18-1, Sakuradai, Minami-ku, Sagamihara, Kanagawa 252-0392 Japan; Department of Pediatrics, Iwakiri Hospital, Miyagi, Japan

**Keywords:** Egg, Food hypersensitivity, Oral food challenge, Milk, Symptom assessment

## Abstract

**Background:**

Oral food challenge test doses are recommended to be performed at least 20 min apart; however, the times of symptom provocation from the start of the oral food challenge have never been compared between different foods. In this study, the durations from the start of the oral food challenge to symptom development in children with egg or milk allergy were compared.

**Methods:**

Thirty-eight and 74 children who had previously passed oral food challenges to 96 g of yogurt and pumpkin cake containing ¼ whole egg underwent oral food challenges with 200 mL raw cow’s milk and 1 whole scrambled egg, respectively; of these, 15/38 and 33/74 children had a reaction.

**Results:**

The median ages of patients with a positive challenge were 5.8 and 5.1 years for milk and eggs, respectively. The median times for the first symptom occurrence were 20 min (range, 5–55 min) and 50 min (5–480 min), respectively (*p* = 0.009). The first symptoms developed within 30, 60, and 90 min in 12/15 (80 %), 15/15 (100 %), and 15/15 (100 %) children with milk allergies, and in 10/33 (30.3 %), 20/33 (60.6 %), and 26/33 (78.8 %) children with egg allergies, respectively. The median times of peak symptoms were 50 min (10–210 min) and 120 min (30–560 min) (*p* = 0.001), and those of symptom disappearance were 90 min (30–240 min) and 180 min (80–700 min) for milk and eggs (*p* = 0.002), respectively.

**Discussion:**

Based on the results of our study, symptoms developed within 30 min for only a subset of patients for eggs, and may even take upwards of 60 min to develop. The times of symptom disappearance were 90 min and 180 min for milk and eggs, respectively, indicating that egg-allergic patients should be observed for a longer period time than milk-allergic patients.

**Conclusions:**

Allergic reactions induced by milk appeared and disappeared sooner than those induced by eggs.

**Electronic supplementary material:**

The online version of this article (doi:10.1186/s40413-016-0104-5) contains supplementary material, which is available to authorized users.

## Background

Oral food challenge (OFC) tests are the gold standard in food allergy diagnosis [1, 2]. The PRACTALL guidelines [3], an international study discussing the best practices for OFCs, recommend an interval of at least 20 min between challenge doses; however, the times of symptom provocation from the start of the OFC have never been compared between different foods. In one previous study, an OFC was conducted with peanuts at 2-h intervals, and the median time of symptom appearance was found to be 55 min [4]. In general, OFCs for egg and milk are performed at 30-min intervals [5]; however, it is unclear whether an observation time of 30 min is sufficient [6, 7], and the only way to confirm this is to perform the OFC in one single administration [8]. To address these issues, this study aimed to compare the durations from the start of the OFC to symptom development between children with egg and milk allergies.

## Methods

### Study population

This study was a retrospective chart review of OFCs performed from 2010 to 2011 at the Sendai Medical Center in Japan. Patients who were strongly suspected to have egg or milk allergy, such as those who had experienced an allergic reaction to egg or milk, including eczema, that improved by elimination of egg or milk, or who were positive for egg white-specific immunoglobulin (Ig) E or milk-specific IgE, were subjected to an OFC. Children with milk allergy who had previously passed an OFC with 96 g of yogurt (equal to 100 mL of milk) were given 200 mL of raw milk (milk protein, 6800 mg), and those with suspected egg allergy who had passed an OFC of 1/4 whole egg pumpkin cake (egg protein, 1550 mg) were given 45 g of scrambled egg (egg protein, 6200 mg). The scrambled eggs were cooked by skillet (60 g of raw egg, 1 min, 150 °C). Patients who reacted to 200 mL of milk or 45 g of scrambled egg were the target patients of this study. Hence, we excluded the patients who passed the tests with 200 mL of raw milk or 45 g of scrambled egg, and patients whose clinical data such as age, sex, history of immediate reaction, and allergic complications, or laboratory data such as antigen-specific IgE, were missing.

### Oral food challenge test

OFCs with 200 mL raw cow’s milk and 45 g of scrambled hen’s egg were performed. As mentioned above, the scrambled egg was briefly fried over high heat for 1 min. The raw milk or scrambled egg was administered all at once. The OFC was performed by an open challenge method during hospitalization. Appropriate measures, including fluid resuscitation, oxygenation, antihistamine and steroid administration, β_2_ stimulant inhalation, and adrenaline injections, were applied based on the severity of symptoms according to the anaphylaxis guidelines in Japan (See Additional file 1: Table S1). The severity score was assessed based on the organ system most affected. In children who reacted to 200 mL of milk or one scrambled egg, we compared the rates of each symptom, the times of symptom appearance, times when the symptoms peaked, and times of symptom disappearance.

OFCs were not performed on patients with comorbid symptoms, such as severe eczema or uncontrolled respiratory issues, deemed to potentially affect the results of the OFC.

### IgE measurement assays

The total IgE, egg white-specific IgE, ovomucoid-specific IgE, and cow’s milk-specific IgE titers (Immuno CAP™; Thermo Fisher Scientific/Phadia, Uppsala, Sweden) were measured within 6 months of the OFC.

### Statistical analysis

The results of the statistical analyses are expressed as the median value and range. To statistically compare the two groups, we used the Mann–Whitney *U* test or Fisher’s exact test, and *p* < 0.05 was considered statistically significant. All data were statistically analyzed using SPSS 20.0 software (IBM Corp., Armonk, NY, USA).

### Ethics, consent, and permissions

According to the Declaration of Helsinki, the study design and risks of symptom provocation were fully explained to the patients and patients’ guardians both verbally and in writing, and written informed consent was obtained from all participants to undergo the OFC. For providing an explanation to the children, we used an illustrated document created by a child life specialist. The authors had direct interactions with all patients during the OFC. The retrospective analysis of the results was approved by the institutional review board of Sendai Medical Center. Personal detailed information of the patients was separated from the results used in this study and all clinical data were completely anonymized prior to the analyses.

## Results

### Patient characteristics and symptoms

To evaluate the timing of symptom occurrence following cow’s milk and hen’s egg exposure, OFCs were performed on 38 and 74 children, respectively; of these, 15/38 and 33/74 children had a reaction. We excluded the patients with negative results (23 and 41 patients from the milk and egg OFCs, respectively), with the remaining 15 milk allergic patients and 33 egg allergic patients representing the matched target of our study. All of these 48 patients had available clinical and laboratory data. The median ages of the patients with a positive challenge were 5.8 and 5.1 years for milk and eggs, respectively (See Additional file 2: Table S2). The sex, history of food allergies, allergic complications (atopic dermatitis, bronchial asthma, allergic rhinitis), and total IgE levels did not significantly differ between the two groups. Further, there were no differences in the severity of symptoms (grade of severity scores) and symptom appearance between the two groups. No circulatory symptoms were seen; however, skin, gastrointestinal, and respiratory symptoms occurred in both groups (See Additional file 3: Table S3). Moreover, there was no difference in the treatments administered for the provoked symptoms.

### Timing of symptoms

**Fig. 1:**
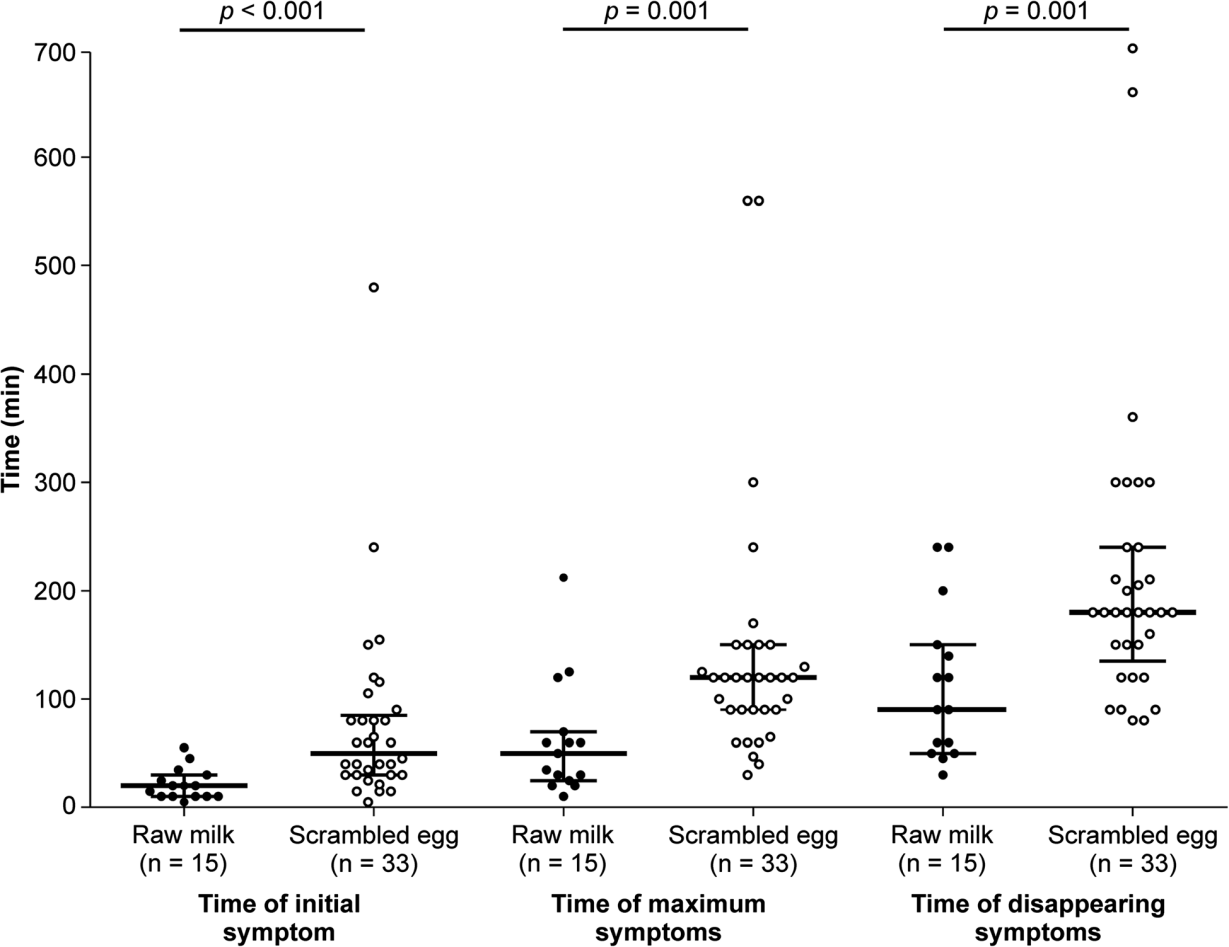
Times of initial symptoms, maximum symptoms, and symptom disappearance after the oral food challenges. Horizontal lines indicate the median (range). *p* values indicate significant differences in the times of symptom appearance/disappearance between the two groups

The median times for the first symptom occurrence were 20 min (range, 5–55 min) and 50 min (5–480 min) for milk and eggs (*p* = 0.009), respectively (Fig. 1). The first symptoms developed within 30, 60, and 90 min in 12/15 (80 %), 15/15 (100 %), and 15/15 (100 %) children with milk allergy and in 10/33 (30.3 %), 20/33 (60.6 %), and 26/33 (78.8 %) children with egg allergy, respectively. The median times of peak symptoms were 50 min (10–210 min) and 120 min (30–560 min) (*p* = 0.001), and the median times of symptom disappearance were 90 min (30–240) and 180 min (80–700) for milk and eggs (*p* = 0.002), respectively. The latest time of the first symptom appearance was 55 min for milk, after which symptoms had appeared in all patients. For eggs, the symptoms appeared in 29/33 patients within 120 min and occurred at a maximum of 480 min. The initial skin and respiratory symptoms for milk developed earlier than those for eggs (Table 1). The times of maximum symptoms and symptom disappearance for the skin, respiratory system, and gastrointestinal tract were all earlier in children with milk allergy than in children with egg allergy. There was no difference between the patients who received and did not receive treatment in the time of peak symptom and disappearance in this study.

**Table 1:** Times of skin, gastrointestinal, and respiratory symptoms induced by the oral food challenge

Food	Raw milk (*n* = 15)	Scrambled egg (*n* = 33)	*p* value
Skin	(*n* = 12)	(*n* = 22)	
Time of initial symptom	25 (5–60)	58 (5–480)	0.003
Time of maximum symptom	55 (20–210)	120 (30–560)	0.02
Time of symptom disappearance	90 (45–240)	180 (70–700)	0.02
Gastrointestinal	(*n* = 7)	(*n* = 23)	
Time of initial symptom	20 (2–60)	40 (5–240)	0.13
Time of maximum symptom	25 (8–70)	90 (30–300)	0.03
Time of symptom disappearance	50 (10–150)	150 (60–360)	0.04
Respiratory	(*n* = 9)	(*n* = 15)	
Time of initial symptom	20 (5–55)	55 (15–120)	0.004
Time of maximum symptom	40 (8–70)	90 (20–150)	0.01
Time of symptom disappearance	60 (10–120)	150 (30–180)	0.02

*p* < 0.05 was considered statistically significant

Data are expressed as the median (range) times (in minutes)

## Discussion

### Timing of symptoms

To the best of our knowledge, this is the first study to compare the times of symptom development between eggs and milk. Allergic reactions induced by milk appeared sooner than those induced by eggs. Based on the results of our study, symptoms developed within 30 min for only a subset of patients for eggs, and may even take upwards of 60 min to develop. The times of symptom disappearance were 90 min and 180 min for milk and eggs, respectively, indicating that egg-allergic patients should be observed for a longer period time than milk-allergic patients.

### Timing between doses of an oral food challenge test

Blumchen et al. [4] reported on multiple incremental dose increases, and concluded that threshold dose challenges may need longer periods of time between doses compared to regular OFCs. Similarly, our previous study also suggested that the intervals between challenges should be longer than those of regular OFCs [9]. In determining the appropriate threshold doses, the timing between doses matters greatly [6]. Our study participants had been previously determined to have relatively high thresholds by prior OFCs, and one single large-dosed challenge could hence be implemented safely. Although this study cannot be generalized to all food allergy patients, one single-dose administration may be applicable when OFCs have been previously performed, as in the present study.

### Why do allergic reactions to milk appear sooner than reactions to hen’s eggs?

In this study, for hen’s eggs, the symptoms developed after an ample time had passed. To our knowledge, the blood concentration of ovalbumin has never been reported. However, it has been well established that egg whites contain protease inhibitors, including ovomucoid, ovoinhibitor, and ovomacroglobulin, which may be hard to digest during early childhood, owing to the function of the digestive enzymes being insufficient, particularly in patients with food allergies [10]. Therefore, the absorption is delayed, and the time that the antigen stays in the intestine increases, consequently resulting in digestive tract symptoms potentially developing. On the other hand, as milk is a liquid, absorption occurs early. The blood concentration of milk reaches a peak at 1 h after ingestion and decreases by half in 4 h as a result of its fast absorption [11], and this might be the reason for why the symptoms develop earlier. Nevertheless, further studies will be needed to reveal the exact mechanism of this phenomenon.

### Limitations

Our study revealed that allergic reactions to milk appear sooner than reactions to hen’s eggs for the first time.

However, this study has certain limitations. First, the number of patients in this study was limited. Second, this study cannot be generalized to all egg and milk allergic patients. The study participants had all passed previous OFCs to either 96 g of yogurt or baked egg prior to being challenged, and it is possible that more sensitive patients may react more quickly than these patients. Moreover, children allergic to milk and egg in the general population tend to be aged <5 years [3], whereas the median age of the patients included in this study was more than 5 years. Third, this study was an open challenge test and not a double-blind placebo-controlled OFC; hence, additional studies to further confirm our results are warranted.

## Conclusion

We here compared the symptom timing after egg and milk OFCs. This is the first report to compare the duration from the start of an OFC to symptom development in children with egg or milk allergy. The allergic reactions induced by milk both appeared and disappeared sooner than those in response to hen’s eggs. Although the subjects were selected and the results are thereby hard to generalize, our findings indicate that the intake interval for OFCs may require a longer time for eggs than for milk. Thus, to assess the provoked symptoms upon milk or egg exposure, egg-allergic patients should be observed for a longer duration than milk-allergic patients. These findings will contribute to improving the safety of OFCs and the observation of induced symptoms by different foods.

### Ethics approval and consent to participate

According to the Declaration of Helsinki, the study design and risks of symptom provocation were fully explained to the patients and patients’ guardians both verbally and in writing, and written informed consent was obtained from all participants to undergo the OFC. For providing an explanation to the children, we used an illustrated document created by a child life specialist. The authors had direct interactions with all patients during the OFC. The retrospective analysis of the results was approved by the institutional review board of Sendai Medical Center. Personal detailed information of the patients was separated from the results used in this study and all clinical data were completely anonymized prior to the analyses.

## Additional files

Additional file 1: Table S1. Grading symptoms. (XSLX 11kb) Additional file 2: Table S2. Clinicodemographic features of the study patients. (XSLX 10kb) Additional file 3: Table S3. Data related to induced reactions and treatments for positive challenges. (XSLX 10kb)

## Abbreviations

Ig: immunoglobulin
OFC: oral food challenge
